# A first report of progressive multifocal leukoencephalopathy in childhood-onset NMOSD

**DOI:** 10.1177/13524585251331855

**Published:** 2025-04-18

**Authors:** Pakeeran Siriratnam, Simon Gosling, Maneesh Bhojak, Michael Griffiths, Rachel Kneen, Evangeline Wassmer, Saif Huda

**Affiliations:** Department of Neurology, The Walton Centre NHS Foundation Trust, Liverpool, UK; Department of Neuroscience, Central Clinical School, Monash University, Melbourne, VIC, Australia; Department of Neurology, Birmingham Women and Children’s Hospital, Birmingham, UK; Department of Neuroradiology, The Walton Centre NHS Foundation Trust, Liverpool, UK; Centre of Child and Adolescent Health research, The University of Sydney, Camperdown, NSW, Australia; Department of Neurology, Alder Hey Children’s NHS Foundation Trust, Liverpool, UK; Department of Neurology, Birmingham Women and Children’s Hospital, Birmingham, UK; Department of Neurology, The Walton Centre NHS Foundation Trust, Liverpool, UK

**Keywords:** Neuromyelitis optica (NMO), immunology

## Abstract

**Background:**

Progressive multifocal leukoencephalopathy (PML) has not been reported in pediatric neuromyelitis optica spectrum disorder (NMOSD) and rarely described in children.

**Objectives:**

To report a case of PML in childhood-onset NMOSD.

**Methods:**

A single retrospective case report.

**Results:**

Nine years after diagnosis of aquaporin-4 antibody positive NMOSD, a 17-year-old boy treated with rituximab presented with painless right visual loss over 6 weeks coinciding with CD19 repopulation. Acute relapse treatment was given but he continued to deteriorate, and JC virus was detected in cerebrospinal fluid confirming PML.

**Conclusion:**

PML can occur in childhood-onset NMOSD and protracted clinical presentations with unusual radiological features should prompt JCV testing. Balancing effective immunosuppression while mitigating the risks of associated complications in long-term relapsing conditions remains a challenge.

## Background

Neuromyelitis optica spectrum disorder (NMOSD) is a rare autoimmune disease associated with aquaporin-4 antibodies (AQP4-IgG). Disease onset in childhood accounts for 3%–5% of all cases.^1^ High-efficacy immunotherapies like rituximab (anti-CD20 chimeric monoclonal antibody; off license) are commonly used to reduce NMOSD relapse risk, but complications such as secondary hypogammaglobulinemia, neutropenia, and infections, including progressive multifocal leukoencephalopathy (PML), can occur. PML is a rare infection of oligodendrocytes caused by reactivation of the human polyomavirus JC virus (JCV).^2^ While PML was originally described in the context of hematological malignancies and advanced human immunodeficiency virus (HIV) disease, there are increasing reports of PML secondary to immunotherapies such as natalizumab and, much less frequently, rituximab.^2,3^ To our knowledge, this is the first reported case of PML in childhood-onset NMOSD.^4^

## Case report

**Figure 1.**
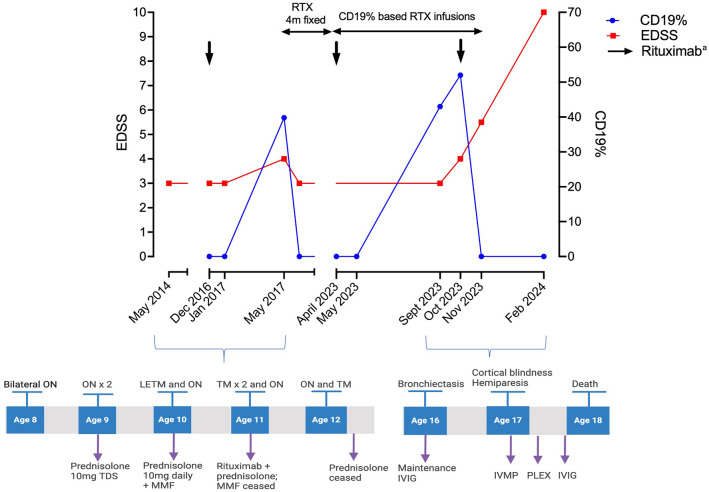
Treatment and relapses. The patient initially presented with bilateral optic neuritis at age 8 and experienced two optic neuritis relapses the following year. NMOSD was confirmed and he was treated with prednisone 30 mg/day. After further optic neuritis and myelitis relapses, MMF was added. Further episodes of myelitis and optic neuritis prompted a treatment change to RTX (black solid arrows). The development of bronchiectasis and hypogammaglobulinemia necessitated the commencement of monthly IVIG. ON: optic neuritis; TM: transverse myelitis; LETM: longitudinally extensive transverse myelitis; MMF: mycophenolate mofetil; RTX: rituximab; IVIG: intravenous immunoglobulin; PLEX: plasma exchange; EDSS: Estimated Disability Status Scale, PBMC: peripheral blood mononuclear cells; NMOSD: neuromyelitis optica spectrum disorder. ^a^Not all RTX infusions are shown. Initially, a fixed 6-month dosing schedule was planned. However, due to a relapse (sequential optic neuritis and myelitis) at approximately 4 months linked to CD19% repopulation, the regimen was adjusted to fixed 4-monthly RTX infusions. In the last 2 years, variable dosing based on CD19% levels (re-treatment threshold at 1% of PBMCs) was implemented. BioRender.

A 17-year-old right-handed boy with a 9-year history of AQP4-IgG NMOSD presented with painless and progressive visual deterioration over 6 weeks. He was diagnosed at the age of 8 years following a presentation with bilateral optic neuritis (Figure 1). After experiencing multiple relapses on mycophenolate mofetil and prednisolone, his treatment was changed to intravenous rituximab 2.2 g per infusion (1.1 g administered 2 weeks apart). However, he relapsed 4 months after the initial infusion, which coincided with CD19^+^ B-lymphocyte repopulation. Consequently, his treatment was adjusted to 4-monthly dosing with intermittent CD19^+^ B-cell monitoring, consistently showing repopulation at 4–5 months. To reduce the immunosuppression burden on adult neurology service transition, his treatment was modified 2 years prior to presentation. Monthly monitoring was commenced with 1 g per re-infusion based on a 1% CD19 threshold. Although he remained relapse-free for over 5 years, he developed secondary hypogammaglobulinemia approximately 6 years after rituximab initiation, with recurrent lower respiratory tract infections and bronchiectasis. He also had recurrent cutaneous herpes simplex and warts since age 3. Baseline CD4 counts were unavailable; however, his CD4 count a year prior to the current presentation was 37 cells/μL (normal range: 500–1500), and his lymphocyte count was 0.78 × 10⁹/L (normal range: 1–5 × 10⁹/L) prior to rituximab initiation. HIV screening, trio whole genome sequencing, and primary immunodeficiency screening (Health Genomics England (R 15.4)) were negative. Prophylactic antibiotics and intravenous immunoglobulin 25 g every 3 weeks were started and a switch to anti-interleukin 6 therapy was planned.

Prior to his current presentation, visual acuities were right; 20/15 and left; hand movements. The remainder of his neurological examination was unremarkable. At presentation with bilateral visual loss (right eye: 20/80 and left eye: light perception), his CD19+ B lymphocytes were elevated to 43% of peripheral blood mononuclear cells. He was treated with intravenous methylprednisolone (1 g/day for 5 days) followed by rituximab (1 g) and an oral prednisone taper. However, repeat neurological examination 1 month later demonstrated cortical blindness, right-sided pyramidal weakness (MRC score 0/5 arm and 4/5 leg), and cognitive slowing, without hemianopia, ataxia, or seizures. Magnetic resonance imaging (MRI) showed bilateral occipital and temporal T2 signal change (Supplementary Figure 1a–d). Intravenous methylprednisolone, five cycles of plasma exchange, and intravenous immunoglobulin were administered. Progressive multifocal hyperintense T2 signal changes were observed, with peripheral diffusion restriction but no post-contrast enhancement (Supplementary Figure 1e–g). His CD4^+^ T-cell and total lymphocyte counts were 26 (500–1500) cells/μL and 0.24 × 10⁹/L (1–3 × 10⁹/L), respectively. Differentials included atypical infections and malignancies. Cerebrospinal fluid (CSF) cell and protein count were normal, but CSF JCV PCR was positive (>100,000 copies), confirming PML.^5^ He died 3 months from symptom onset and 40 days after PML diagnosis.

## Discussion

We report the first case of PML in childhood-onset NMOSD, with only one prior report of PML in an elderly NMOSD patient.^4^ The rarity of PML in NMOSD is likely attributable to both the low prevalence of NMOSD and the avoidance of immunotherapies with a higher association with PML, such as natalizumab.^3^ The reported incidence of PML with rituximab is approximately 1 in 32,000; and to date, only one case of PML has been reported with rituximab monotherapy in multiple sclerosis.^3,6^

The rarity of PML in NMOSD, frequency of cerebral lesions in childhood-onset NMOSD (16%–32%), presence of CD19+ B-cell repopulation and awareness that relapses on treatment may present in a more protracted manner made this a challenging diagnosis.^1^ While earlier CSF JCV PCR examination may have resulted in a faster diagnosis, a negative CSF JCV PCR does not exclude the diagnosis and brain biopsy may be required.^7^ Although 12-month survival in PML secondary to natalizumab-associated PML is over 75%, evidence in rheumatological conditions treated with rituximab suggest median time to death after diagnosis of 2 months, with a case fatality rate of 90%, in keeping with the rapid progression seen in our case.^3,8^ Currently, there are no established treatments for PML, and experimental therapies including adoptive transfer of polyomavirus-specific T cells were considered but not given due to the risk of immune reconstitution syndrome, rapid neurological deterioration and preference of the patient and family.^7^

Several factors likely contributed to the development of PML in this patient. Rapid B-cell re-population required frequent re-dosing of rituximab. Despite his complications, de-escalation of rituximab or extending dosing intervals with B-cell repletion poses a significant risk of relapse.^9^ Furthermore, alternative monoclonal antibody therapies were not available in the National Health Service (NHS). Testing for FCGR3A polymorphisms was unavailable but may have explained rapid B-cell re-population. In addition, the cumulative impact of prior immunotherapies, including prednisolone and mycophenolate mofetil, were likely contributors. Despite negative genetic testing for primary immunodeficiency syndromes, this remained a suspicion given the low CD4^+^ T-cell count and severe recurrent infections. Prior studies have identified that the disease onset is contingent upon a decline in JCV-specific cellular immunity, with CD4^+^ T-cell count correlating with PML prognosis.^10^ Although CD4 counts were not tested before rituximab initiation, a progressive decline in CD4^+^ T-cell and total lymphocyte count was observed. The pathophysiology underlying rituximab-associated PML or reduction in T lymphocytes remains unclear. While T cells can express CD20, and peripheral blood CD4^+^ T-cell reductions have been observed in NMOSD patients, such profound depletion would be unexpected.^3^

In summary, although rare, PML should be considered in NMOSD patients receiving rituximab, especially in cases of protracted relapse presentations with atypical radiological findings. This case underscores the challenges of balancing effective immunosuppression while mitigating associated risks in relapsing neurological conditions. Further research is needed to better understand the mechanism of rituximab-induced PML and improve management strategies.

## Supplemental Material

sj-docx-1-msj-10.1177_13524585251331855 – Supplemental material for A first report of progressive multifocal leukoencephalopathy in childhood-onset NMOSD Supplemental material, sj-docx-1-msj-10.1177_13524585251331855 for A first report of progressive multifocal leukoencephalopathy in childhood-onset NMOSD by Pakeeran Siriratnam, Simon Gosling, Maneesh Bhojak, Michael Griffiths, Rachel Kneen, Evangeline Wassmer and Saif Huda in Multiple Sclerosis Journal
