# Supplementary material for: A first report of progressive multifocal leukoencephalopathy in childhood-onset NMOSD
Source: Mult Scler. 2025 Apr 18;31(13):1604–7. doi: 10.1177/13524585251331855 (PMC12589659; doi:10.1177/13524585251331855)
Supplement: sj-docx-1-msj-10.1177_13524585251331855 – Supplemental material for A first report of progressive multifocal leukoencephalopathy in childhood-onset NMOSD [file sj-docx-1-msj-10.1177_13524585251331855.docx]

**^Supplementary Figure 1.^** ^Magnetic resonance imaging of the brain.^


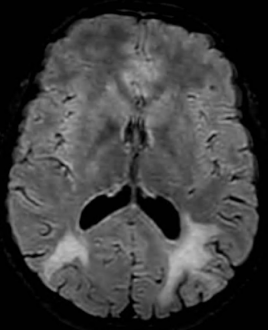


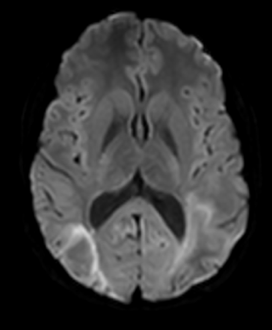

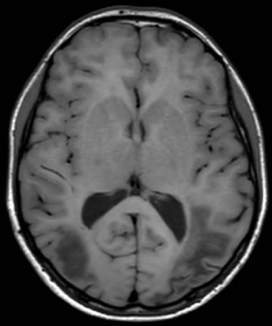
^
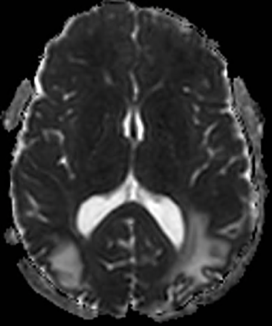
^

d

c

b

a


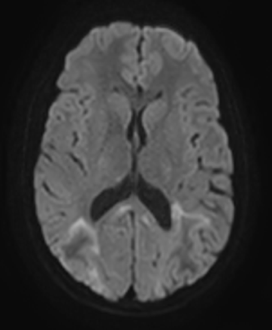

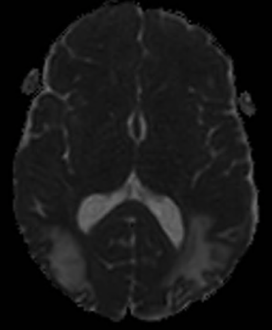

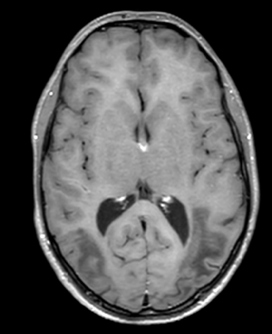

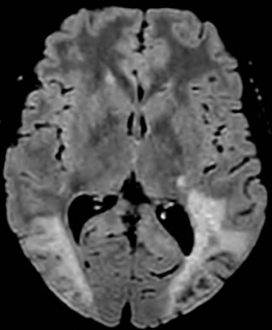


h

g

f

e

^Top row (a-d)- MRI at presentation with visual disturbance (e-h)- Re-imaging after steroids, intravenous immunoglobulin and plasma exchange. (a) Axial FLAIR sequence showing widespread T2 white matter changes in the temporo-occipital regions, more extensive in the left hemisphere (motion-degraded image); (b) Diffusion weighted imaging (DWI) shows peripheral diffusion restriction in the lesion; (c) Apparent diffusion coefficient (ADC) demonstrates corresponding areas of diffusion restriction; (d) Post-contrast T1 axial imaging showing absence of contrast enhancement; (e) Axial FLAIR image showing progression of temporo-occipital T2 signal change; (f) DWI showing peripheral restricted diffusion as before; (g) ADC shows corresponding diffusion restriction; (h) post-contrast T1 axial imaging showing absence of contrast enhancement.^
